# Systematic review of cognitive impairment in drivers through mental workload using physiological measures of heart rate variability

**DOI:** 10.3389/fncom.2024.1475530

**Published:** 2024-10-31

**Authors:** Mansoor S. Raza, Mohsin Murtaza, Chi-Tsun Cheng, Muhana M. A. Muslam, Bader M. Albahlal

**Affiliations:** ^1^STEM College, RMIT University, Melbourne, VIC, Australia; ^2^Department of Information Technology, College of Computer and Information Sciences, Imam Mohammad Ibn Saud Islamic University (IMSIU), Riyadh, Saudi Arabia

**Keywords:** mental workload (MWL), heart rate variability (HRV), take over request (TOR), society of automotive engineers (SAE), driver cognitive impairment, human machine interface (HMI), eye tracking, driver safety

## Abstract

The intricate interplay between driver cognitive dysfunction, mental workload (MWL), and heart rate variability (HRV) provides a captivating avenue for investigation within the domain of transportation safety studies. This article provides a systematic review and examines cognitive hindrance stemming from mental workload and heart rate variability. It scrutinizes the mental workload experienced by drivers by leveraging data gleaned from prior studies that employed heart rate monitoring systems and eye tracking technology, thereby illuminating the correlation between cognitive impairment, mental workload, and physiological indicators such as heart rate and ocular movements. The investigation is grounded in the premise that the mental workload of drivers can be assessed through physiological cues, such as heart rate and eye movements. The study discovered that HRV and infrared (IR) measurements played a crucial role in evaluating fatigue and workload for skilled drivers. However, the study overlooked potential factors contributing to cognitive impairment in drivers and could benefit from incorporating alternative indicators of cognitive workload for deeper insights. Furthermore, investigated driving simulators demonstrated that an eco-safe driving Human-Machine Interface (HMI) significantly promoted safe driving behaviors without imposing excessive mental and visual workload on drivers. Recommendations were made for future studies to consider additional indicators of cognitive workload, such as subjective assessments or task performance metrics, for a more comprehensive understanding.

## 1 Introduction

Mental workload (MWL) within the scope of driver cognitive impairment refers to the mental effort required for the performance of driving-related activities and the subsequent impacts of this effort on a driver's cognitive abilities. It includes the demands placed on a driver's cognitive resources by the driving environment and associated tasks, such as maneuvering through traffic, reacting to traffic control signage, and operating the vehicle's controls. An increased mental workload may trigger cognitive deficits, thereby undermining a driver's ability to process information, make decisions, and react appropriately to diverse driving situations. This scenario may result in reduced driving performance and an elevated risk of traffic accidents. Factors influencing mental workload comprise the complexity of the driving environment, the driver's level of experience, and the presence of external distractions (Paxion et al., 2014; Chen and Terken, 2022). Driver safety holds significant importance due to its direct influence on the wellbeing of road users and overall public safety. Cognitive impairment is a crucial aspect when considering safe driving, as it can hinder an individual's capacity to process information, make prompt decisions, and react efficiently to ever-changing traffic scenarios. Recent studies have emphasized the vital significance of MWL and heart rate variability (HRV) in shaping the cognitive performance of drivers. MWL pertains to the cognitive challenges faced by drivers during different driving tasks, such as maneuvering through complex intersections or dealing with distractions on the road. Concurrently, HRV, which mirrors the adaptability of the autonomic nervous system, impacts the physiological responses to stressors and cognitive demands while driving (Kanakapura Sriranga et al., 2023; Lohani et al., 2019). It is imperative to comprehend the intersection of MWL and HRV within the driving context to develop precise interventions, improve driver training schemes, and elevate road safety standards. The manuscript presents a comprehensive review and in-depth examination of cognitive impairments linked to mental workload and heart rate variability. It analyzes the multifaceted relationship between cognitive deficiencies, mental workload, and physiological measures like heart rate. Through the analysis of this interaction, the research seeks to evaluate the mental workload encountered by drivers utilizing data derived from heart rate monitoring systems utilized in earlier investigations. The results possess the capacity to substantially impact the advancement of innovative technologies aimed at mitigating cognitive impairments induced by mental workload. The research methodology employed in the paper is distinguished by its methodical and rigorous approach, as it integrates the Scanning, Predicting, Identifying, Deciding, and Executing a Response (SPIDER) and Preferred Reporting Items for Systematic Reviews and Meta-Analyses (PRISMA) frameworks for the purpose of collating and assessing pertinent literature (Peixoto et al., 2021; Cooke et al., 2012). The discoveries stemming from the study enrich the comprehension of cognitive impairments linked to mental workload and heart rate variability, furnishing valuable insights that can enhance driver safety and facilitate the development of efficacious interventions. This paper presents a review and analysis of cognitive impairment caused by MWL and HRV.

## 2 Literature review

This literature review focused on cognitive impairment, mental workload, and physiological indicators like heart rate in drivers. The reviewed studies can be categorized into three main areas:

- Adaptive interfaces and driver assistance systems in Cognitively Challenging Situations;
- Driver mental workload assessment techniques;
- Relationship between physiological measures and driver cognitive states.

### 2.1 Adaptive interfaces and driver assistance systems in Cognitively Challenging Situations

Several studies have explored the development and evaluation of adaptive interfaces and driver assistance systems to support drivers during cognitively demanding situations. An adaptive multi-modal interface model was developed to assist drivers during a take-over request (TOR) from autonomous to manual driving, focusing on balancing convenience with safety risks (Chen et al., 2021).

A comprehensive evaluation method for highly automated driving functions across Society of Automotive Engineers (SAE) levels 2 to 4 was proposed by Rösener et al. (2017). Notably, human involvement is still requisite up to Level 4 of automation (Murtaza et al., 2023b). This method integrates technical, user-related, in-traffic, and impact assessment components. It uniquely combines various testing tools and methodologies, including field tests, simulations, and user trials, to provide a holistic view of the multifaceted implications of automated driving. The cognitive and emotional impacts on drivers interacting with automated driving technologies have not been incorporated (Rösener et al., 2017).

The effects of haptic assistive driving systems on driving behaviors studied that highlighted the significance of a strong model-free controller for accommodating different driving styles. They ignore the high degree of uncertainties in human behavior. The impact of ambient lighting on driver performance and mental exertion during ten TORs have improved drivers' take-over performance. The sample collected with majority of young women may limiting the generalization (Noubissie Tientcheu et al., 2022).

How behavioral measures impact the braking performance of older drivers under cognitive workloads, particularly focusing on the left foot position as a preventive strategy were studies and, comparing simulated and real road environments to analyze braking errors and predict adaptive skills at intersections, achieving a 70% accuracy in forecasting adaptive skills by assessing the left foot posture of elderly drivers, enhancing the system's effectiveness and usefulness (Figalová et al., 2022). The study revealed that cooperative control strategy for lane-keeping enhanced cooperative driving quality by 9.4% and minimized conflict by 65.38% compared to the previous design without a sharing parameter, along with an 86.13% reduction in steering workload (Kajiwara and Murata, 2022).

They examined the acceptance of advanced safety features in Toyota Sienna and Prius models among 183 vehicle owners through telephone interviews, revealing a high level of interest in adaptive cruise control and forward collision avoidance, but slightly lower acceptance for lane departure warning and prevention, with variations observed based on drivers' age and gender, indicating potential differences in responses to emerging crash avoidance technologies in various vehicle models (Perozzi et al., 2022).

Their study focused on cognitive attributes impacting SA in driving scenarios, identifying eight cognitive states that influence drivers' SA. Their proposed guidelines show significant improvements in SA and decision-making through the new interface compared to existing systems (Eichelberger and McCartt, 2015). The research delves into the effects of training on drivers' capacity to engage with Advanced Driver Assistance Systems (ADAS) and Autonomous Vehicle (AV) technology, showcasing notable enhancements in precision, response time, and vehicle management post-training (Park et al., 2020). The research conducted by Murtaza et al. (2023a) and Murtaza et al. (2024a) underscored the significance of training in mitigating mental workload for operators of Advanced Driver-Assistance Systems (ADAS) and Autonomous Vehicles (AV). The studies compared the efficacy of video-based and text-based training modalities (Murtaza et al., 2024a) and evaluated conventional methods against those based on Large Language Models (LLM) (Murtaza et al., 2024b). These findings emphasize the necessity of designing tailored training programs to ensure the safe and proficient use of these technologies. The cited studies significantly highlight the imperative motivation behind the enhancement of driver safety and overall performance, which can be accomplished through the implementation of innovative adaptive technologies that utilize a variety of methodologies; these include, but are not limited to, the utilization of sophisticated driving simulators, the integration of haptic assistive driving systems, and the application of subjective workload assessments to gauge the efficacy of these interventions. In this context, it would be particularly beneficial to investigate and analyze how the findings from these studies could be further enriched and substantiated by the strategic incorporation of physiological measures that could provide deeper insights into the drivers' responses and behaviors during simulated driving tasks.

### 2.2 Driver mental workload assessment techniques

Researchers have investigated various methods to assess drivers' mental workload, including subjective measures, physiological indicators, and performance metrics. They reviewed the increasing popularity of remote eye measurements as tools for evaluating driver workload without causing distraction, emphasizing the importance of using multiple assessment methods for increased validity and reliability (Marquart et al., 2015). Researcher examined the effectiveness of eye-tracking metrics in evaluating a driver's cognitive load during semi-autonomous driving while performing non-driving tasks. They identified pupil diameter change, saccade characteristics, fixation duration, and 3D gaze entropy as consistent indicators of cognitive load in both visual and auditory multitasking situations (Chen et al., 2022). Their research found the impact of Non-Driving Related Task (NDRT) on takeover performance in Highly Automated Driving (HAD) contexts and the influence of workload on driver takeover performance. The study lacks details on sample size/participants, limiting generalizability (Yoon and Ji, 2019). Their study aims to analyze the relationship of driver reaction time, gaze-on time, road-fixation time, and take-over time. They solely examine the driver's reaction time, disregarding driving accuracy or safety (Ko and Ji, 2018). The study analyzed descriptive statistics and Friedman test results of pupil size under different HMI conditions at stop-sign intersections with and without traffic (Li et al., 2020). In summary, these studies demonstrate the motivation to develop reliable and non-intrusive methods for assessing driver mental workload, employing techniques such as eye tracking, subjective questionnaires, and secondary task performance. However, limitations include the need for more diverse driving scenarios and the potential influence of individual differences on workload measures.

### 2.3 Relationship between physiological measures and driver cognitive states

Various studies have focused on the link between physiological indicators, particularly HRV and visual movements, and the cognitive states of drivers, involving mental workload, fatigue, and drowsiness. One particular study scrutinizes the application of in-vehicle physiological sensors to assess drivers' mental workload, focusing specifically on cardiovascular and respiratory data. This research delineates alterations in driving tasks that affect mental workload and the driver's capacity to regain control of the vehicle (Kanakapura Sriranga et al., 2023). Expanding upon this groundwork, an additional study introduces a methodology that leverages mutual information derived from EEG and vehicular signals to formulate a feature set for evaluating drivers' mental workload. This feature set exhibits promise in forecasting and classifying tasks with minimal error and substantial accuracy, with the Support Vector Machine (SVM) classifier outperforming alternative classifiers. Nonetheless, this investigation overlooks individual characteristics and experiential factors (Islam et al., 2020). Subsequent research emphasizes the evaluation of drivers' cognitive workload through cognitive assessments and physiological indicators such as heart rate variability and infrared thermal imaging. Machine learning models attained elevated levels of accuracy, although this accuracy may be affected by an increased sample size (Cardone et al., 2022). Another analysis underscores the potential of HRV as a metric for mental workload, indicating that the mean heart rate escalates with heightened demands in secondary tasks, and younger drivers display elevated heart rates compared to their older counterparts (Biondi et al., 2017). A systematic review focusing on the identification of driver fatigue through HRV indicates inconsistencies in HRV metrics between alert and fatigued drivers, underscoring the need for additional research into the relationship between HRV and the causes of fatigue (Lu et al., 2022). In conjunction with this, an article introduces a novel adaptive interface bridging human and machine interaction that facilitates the automatic rerouting of incoming phone calls to voicemail without alerting the driver when the estimated workload exceeds a specified threshold (Piechulla et al., 2003). Regarding methodological innovations, one manuscript evaluates the application of wavelet analysis on heart rate variability signals to differentiate between alert and drowsy driving events, contrasting it with the conventional fast Fourier transform approach. The wavelet-based technique demonstrated superior efficacy in classification performance, achieving an accuracy of 95% through leave-one-out validation (Li and Chung, 2013). Another investigation sought to establish a heart rate monitoring system utilizing an Arduino Kit and a heart rate sensor to evaluate drivers' mental workload and enhance road safety through timely notifications (Makhtar and Sulaiman, 2020). In investigating the effects of mental workload fluctuations on cerebral activity, a study examined military drivers in both combat and non-combat contexts using EEG. Elevated theta EEG power spectrum in the frontal, temporal, and occipital regions during complex scenarios signified increased mental workload. The data were scrutinized to assess the implications of workload on cerebral activity and driving efficacy (Diaz-Piedra et al., 2020). The Taiwan Bus Driver Cohort Study (TBDCS) employed a cohort methodology to examine the predictive capacity of HRV analysis concerning cardiovascular disease risk among occupational drivers over an 8-year duration (Wang et al., 2021). A physiological recognition framework was established utilizing HRV metrics for feature construction and selection, which incorporated the Best Individual N (BIN) search method, Kernel-based Class Separability (KBCS) selection criterion, Principal Component Analysis (PCA), and Linear Discriminant Analysis (LDA) for feature extraction, alongside the k-nearest neighbors (k-NN) algorithm for recognition purposes (Wang et al., 2010). Another investigation focused on the capacity to detect drowsiness through HRV analysis in drivers utilizing a driving simulator, integrating time-domain, frequency-domain, and fractal methodologies, in addition to theta brain wave activity (Mahachandra et al., 2012). Research concerning Automated Valet Parking systems explored the impact of scenario-based explanations on user trust and satisfaction, underscoring the necessity of customized solutions for diverse user demographics to bolster trust, enhance user experience, and mitigate cognitive load (Ma and Feng, 2023). The effectiveness of HRV in recognizing driver drowsiness during operation was also assessed through machine learning methodologies, achieving an accuracy rate of 85% with the random forest classifier, although challenges arising from confounding variables were acknowledged (Persson et al., 2020). Furthermore, a study investigated heart rate variability and perceived workload in simulated driving conditions, particularly within work zones on highways. The results indicated that participants encountered heightened workload in work zone environments and under conditions of increased traffic density, despite heart rate metrics remaining largely unaffected (Shakouri et al., 2018). In aggregate, these investigations underscore the impetus to devise trustworthy and non-invasive methodologies for monitoring cognitive states in drivers, leveraging subjective workload assessments, physiological sensors, and machine learning techniques. Notwithstanding the encouraging advancements, challenges such as the necessity for larger sample sizes, validation in real-world settings, and the consideration of individual variability in physiological responses persist. The integration of these multifaceted approaches and findings could facilitate the development of more effective and adaptive driver monitoring systems, ultimately driver welfare.

## 3 Review methodology

The review methodology employed in this paper combines the utilization of two distinct frameworks, namely the SPIDER framework and the PRISMA qualitative review methodology framework. The SPIDER framework, which stands for Sample population, Phenomenon of Interest, Design of Study, Evaluation, and Research Type, provides a structured approach to organizing and analyzing research studies. On the other hand, the PRISMA framework, which stands for Preferred Reporting Items for Systematic Reviews and Meta-Analyses, offers guidelines for conducting systematic reviews and meta-analyses (Liberati et al., 2009; Cooke et al., 2012). By integrating these two frameworks, the review methodology adopted by the paper ensures a comprehensive and rigorous evaluation of the research articles under investigation. To ensure a representative and diverse Sample population for the review, a total of 120 articles were sourced from various digital libraries. These digital libraries included reputable sources such as the Web of Science (WOS) core collection, ACM, IEEE Xplore, Elsevier, MDPI, Springer, Taylor Francis, and the Arxiv Digital Library. This wide-ranging selection of digital libraries helps to encompass a broad spectrum of research articles related to the topic at hand, thus enhancing the overall validity and generalizability of the review findings. To establish the Phenomenon of Interest a systematic and thorough approach to article inclusion, the study followed a well-defined process. This process involved several stages, including preliminary screening, exclusion of duplicate articles, and evaluation of article eligibility based on specific inclusion criteria.

**Figure 1:**
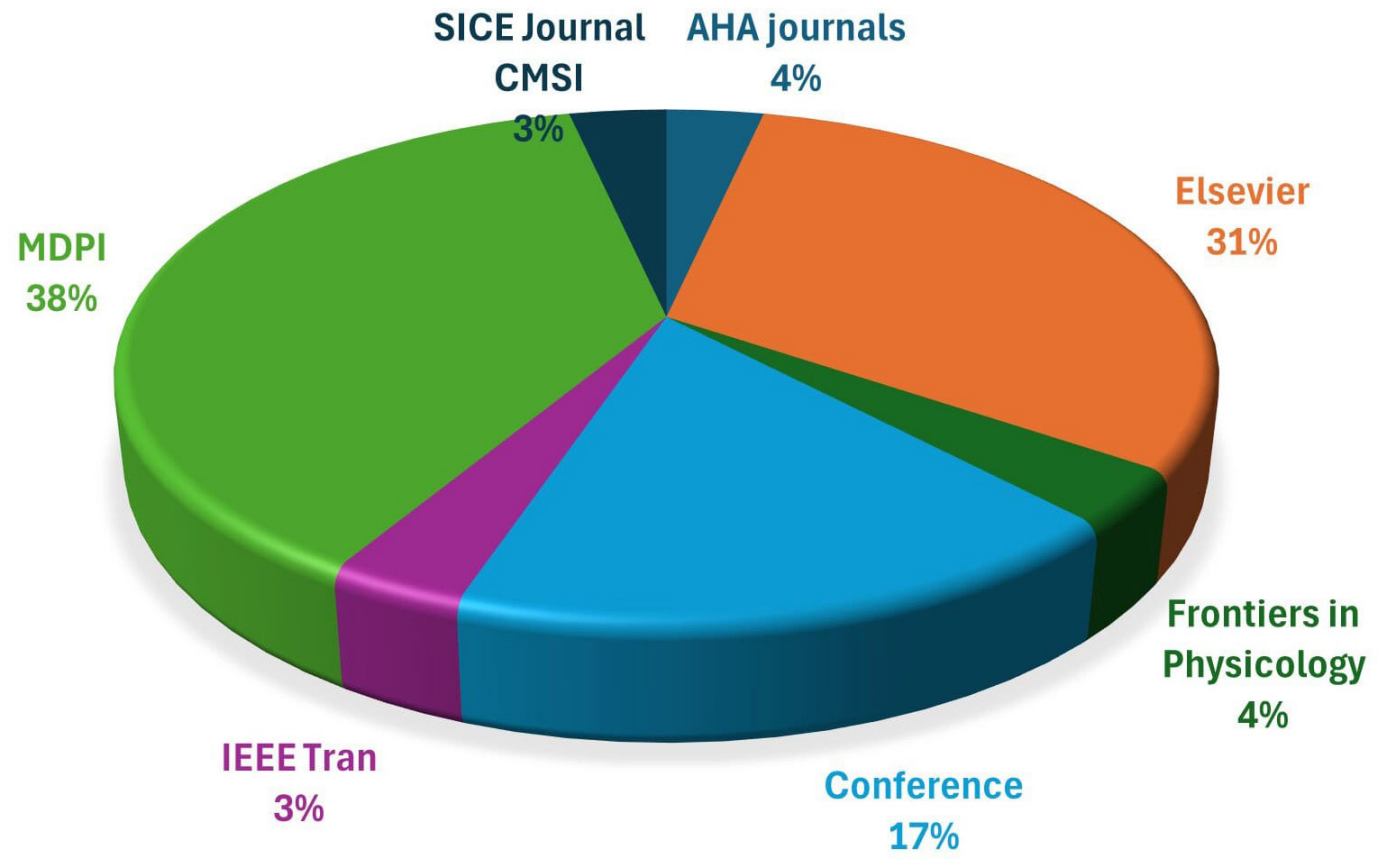
The selected research papers were used in this study.

The Design of study and inclusion criteria were carefully designed to ensure that only relevant and high-quality articles were incorporated into the review. These criteria encompassed factors such as publication in recognized journals or conference proceedings, publication year between 2014 and 2023, relevance to the review objectives, and adherence to established standards. By adhering to these stringent criteria, the review methodology ensures that the selected articles are of high quality and directly contribute to the research objectives. A comprehensive search strategy was devised. To evaluate and identify the relevant articles for review, the strategy involved the use of connecting keywords that were closely aligned with the scope of the review. These keywords included terms such as mental workload, heart rate variability, take over request, society of automotive engineers, driver cognitive impairment and human machine interface. By utilizing these targeted keywords, the review methodology aimed to capture a wide range of articles that are specifically related to driver safety and cognitive impairment. This comprehensive approach ensures that the review encompasses a holistic and in-depth analysis of the topic, thereby enhancing the overall validity and reliability of the review findings. To ensure that the review is not solely reliant on academic literature, the review methodology also incorporated different Research types like gray literature, formal reports, and reputable news repositories. This inclusion of diverse sources of information helps to provide an up-to-date analysis of the trends and developments in MWL, driver safety and cognitive impairment. To identify these additional sources of information, the snowballing method was employed. The snowballing method involves systematically searching through reference lists, citations, and recommendations from existing articles to identify other relevant sources. By utilizing this method, the review methodology ensures that the analysis is comprehensive and reflective of the latest advancements in the field of MWL, driver safety and cognitive impairment. Figure 1 provides insights into the distribution of research papers across different platforms. MDPI stands out as the most prolific publisher, while Elsevier and conference proceedings also play significant roles. These findings contribute to our understanding of scholarly dissemination and academic impact. One article originating from each of the journals Frontiers in Physiology, American Heart Association (AHA), and IEEE Transactions has been included in the study, with each publication contributing to 3% of the total sample. Furthermore, a substantial portion of the literature included in the analysis comes from Elsevier, with a total of nine papers making up 31% of the overall dataset. In addition to journal articles, five papers sourced from conference proceedings were also integrated into the research, accounting for 17% of the total content reviewed. Notably, the highest number of papers, totaling eleven, were obtained from MDPI, making it the most significant contributor to the study with a representation of 38% of the entire dataset. The distribution of literature sources across various publishers and platforms demonstrates a diverse range of scholarly contributions that have been synthesized in this study. This comprehensive approach to selecting sources ensures a robust and well-rounded analysis of the topic under investigation.

## 4 Analysis

HRV, which measures the variation in time between successive heartbeats, has gained significant attention and recognition in the evaluation of MWL. To comprehend the crucial role of HRV, it is essential to look into the concept of MWL itself. When we talk about MWL, we are referring to the cognitive demands that are placed on an individual during the execution of a specific task. This concept encompasses various aspects such as mental effort, attention, and stress. The quantification of MWL plays a pivotal role in optimizing the design of tasks, enhancing overall performance, and ensuring the wellbeing of individuals. HRV Metrics for MWL Assessment: When it comes to the assessment of MWL using HRV, there are various metrics that can be employed. One such set of metrics is the Time Domain Metrics, which involves the analysis of beat-to-beat intervals (RR intervals) over a certain period. Among these metrics, the Standard Deviation of RR Intervals is particularly important as it reflects the overall HRV. Additionally, the Root Mean Square of Successive Differences (RMSSD) is another metric that captures short-term variations in HRV. Moving on to the Frequency Domain Metrics, these metrics focus on analyzing the frequency components of HRV. Low-Frequency (LF) Power is a metric that is linked to sympathetic activity, while the High-Frequency (HF) Power reflects the influence of the parasympathetic branch of the autonomic nervous system. Lastly, the LF/HF Ratio is a metric that provides insights into the autonomic balance. In addition to the above-mentioned metrics, Non-linear Metrics are also used to assess the complexity and adaptability of HRV. These metrics include the Correlation Dimension (D2), which tends to decrease during MWL, indicating reduced adaptability. Furthermore, Sample Entropy (SampEn) is another non-linear metric that measures the irregularity of HRV.

### 4.1 Investigating the relationship between HRV and MWL in driving situations. Part-1

Numerous studies have been conducted to explore the relationship between HRV and MWL. These studies have consistently observed decreased HRV during mental tasks, particularly in the context of non-linear metrics such as D2. Additionally, alterations in HRV have been found to correlate with feelings of frustration and changes in myocardial oxygen consumption. Considering the real-world applications of HRV, it has proven to be a valuable tool in assessing pilot workload, monitoring stress levels in healthcare professionals, and optimizing cognitive performance. By leveraging the insights provided by HRV, individuals and organizations can develop strategies to effectively manage and maintain wellbeing in demanding environments. In this extensive investigation of driving performance, in Table 1 a variety of methodologies have been utilized to uncover crucial insights regarding safety, workload management, and cognitive aspects. The brain's electrical activity serves as a reliable indicator of workload among military drivers, showing significant potential for implementation in practical training situations. The incorporation of wearable sensor technologies, like ear electrode arrays, has the potential to enhance the real-time monitoring of cognitive states, thus improving road safety. Additionally, metrics such as SDNN and LF levels have displayed independent predictive abilities concerning heart and blood pressure conditions, offering valuable information to understand the mental workload in driving situations. The utilization of HRV-based recognition methods has demonstrated superior efficacy compared to other approaches, establishing it as a vital tool for identifying patterns in heart rate variability. Furthermore, biological signals such as RMSSD and SD1 have been recognized as sensitive parameters for detecting sleepiness, showing promise for the advancement of effective sleepiness detection systems. Particularly, explanations based on scenarios have proven to significantly boost situational trust, user experience, and mental workload management among drivers, resulting in quicker reaction times and reduced return times. Moreover, higher situation awareness scores have been linked to more efficient decision-making processes, despite some lingering indecision in decision-making. Interestingly, driving experience has not shown significant impacts on situation awareness or decision-making questionnaire scores. Research has unveiled that ambient in-vehicle lighting can improve drivers' takeover performance, while EEG markers of mental workload have shown no significant differences between experimental and control groups. Furthermore, older drivers have demonstrated increased cognitive errors and mistakes in actions with age progression; however, their accident likelihood has decreased when faced with collision risks. The accurate prediction of coping abilities based on left foot posture near intersections has achieved a commendable accuracy level of 70%. Additionally, evaluations of fatigue and workload levels have heavily relied on heart rate variability and infrared measurements. Adaptive phone settings have reduced mental workload for experienced drivers, with the SVM-based drowsiness detection system outperforming FFT-based methods, achieving an impressive accuracy of 95%. Lastly, heart rate readings during mental workload tasks have provided valuable insights into drivers' physiological reactions. Scholars have delved into methodologies related to heart rate variability and subjective workload in the driving realm, with the random forest classifier emerging as the preferred binary classification tool, despite a 20-percentage point decrease in performance for multi-class classification. Importantly, the development of personalized algorithms is crucial for the efficient monitoring of driver sleepiness based on HRV metrics. Additionally, weak correlations have been noted between heart rate variability measures and subjective workload, as well as between driving performance indicators and subjective workload.

**Table 1:** Analysis of the different methods used to conduct the studies their results and main features.

**References**	**Methods**	**Result**	**Features**
Chen et al. (2021)	DS, AOSPAN, PASAT and NASA-TLX	HFS improved driving, steering wheel operation performance. Reduced response time, mental workload	Heart rate, pupillary response, blink, and eye movement
Rösener et al. (2017)	F&Tt, AdaptIVe-SAE 2–4	There are notable safety consequences between the duration of 2–17 s when the vehicle nears an obstacle in the same lane	The user-related evaluation investigates the interface between user functions, encompassing trust, usability, and acceptance
Noubissie Tientcheu et al. (2022)	LR, HMM, NNM, DBMHGS	Haptic feedback has enhanced driving performance, reduced response time, and lowered MWL	Response time, function, location
Figalová et al. (2022)	DS, F&Tt, EEG, NDRT & TOR	Ambient in-vehicle lighting improves drivers' take-over performance	Ten (TORs) to evaluate the influence of ambient in-vehicle lighting
Kajiwara and Murata (2022)	DS, ML, RF, and NBM	Coping skills can be predicted with 70% accuracy from left foot posture near intersections	Left foot posture, braking
Perozzi et al. (2022)	DS, ADASs, HMI, LKA	LKA improve 9.4% and reduced conflict by 65.38%. Steering workload reduced by 86.13%	Lateral acceleration, road curvature, and MMRDT
Eichelberger and McCartt (2015)	Cohort study, TI	Nine in ten respondents wanted ACC and FCA. 71% wanted LKA. Males were more likely to have LKA than females	ACC, FCA, LKA. MantelHaenszel-Pearson/ Chi-square analysis
Park et al. (2020)	LR, SA, DMQ, ANOVA, cognitive states	There were no significant correlations between driving experience and SA or DMQ scores	Mental model, workload, memory
Murtaza et al. (2023a)	DS, ADAS, RMSE	Vehicle trajectory analysis revealed that training helped minimize serpentine driving behaviors and improve vehicle control	PA, DiR, VLP
Murtaza et al. (2024a)	DS, AV, Sw&AdT	Video-based training yielded better performance outcomes, more accurate mental models, and a deeper understanding of ADAS	LKA, CA, ACC
Marquart et al. (2015)	LR, NASA-TLX, RSME, questionnaires, ISA method	Workload increases, Blink duration and gaze variability decreased	Blinks, fixations, saccades, pupillometry
Chen et al. (2022)	DS, NASA-TLX, MWL, KNN, PCA, PLM classifiers	The accuracy of mental workload K-NN was 88.9%	Eye-tracking metrics
Yoon and Ji (2019)	DS, NASA-TLX, NDRT, Protocol (IRB), TOR	NDRT type has a significant effect on workload	Gaze on time, fixation time, hands-on time, and takeover time
Ko and Ji (2018)	DS, NDRT, TOR, RBs *F*-test, MANOVA	Significant impact of the experimental condition on gaze-on time, road-fixation time, and take-over time, but not on hands-on time	Driver reaction time, gaze-on time, road-fixation time, take-over time
Li et al. (2020)	DS, Friedman test	The HMI for eco-safe driving could promote eco-safe driving behaviors without overburdening drivers	Brake force, acceleration, Eye blinks, pupil size
Kanakapura Sriranga et al. (2023)	LR, PRISMA, MWL	HR and HRV were found to be more sensitive to changes in MWL, while LF & HF components of HRV were considered better indicators	HR, HRV, RR
Islam et al. (2020)	F&Tt, EEG, VS, ML & MWL score	SVM performed the best in both MWL and event classification tasks, achieved a high accuracy of 94%	EEG and vehicular signals
Cardone et al. (2022)	DS, ML, ECG, HRV, HRV+IR thermal imaging	IR + HRV accuracy 73.1%, DST 75%	MWL, HRV, IR
Biondi et al. (2017)	F&Tt, HRV, MWI, DRT, NASA-TLX, IVIS, OSPAN	Younger drivers showed a larger increase in heart rate compared to older drivers. AHR was higher during the OSPAN task	Average heart rate (AHR)
Lu et al. (2022)	LR, HRV, alert and fatigued drivers	HRV-based fatigue detection accuracy 44%–100%	HRV, EEG, N1 sleep stage, fatigue
Piechulla et al. (2003)	F&Tt, ECG, EMG, NASA-TLX	Reduction of MWL in adaptive telephone condition for experienced drivers	ECG, EMG, HRV
Li and Chung (2013)	DS, HRV, FFT, ROC, LOO, SVM	The SVM performance of 95% accuracy, sensitivity and specificity outperforming the FFT-based results	Drowsiness, alert, age, sex, heart disease, hypertensive
Makhtar and Sulaiman (2020)	Arduino Kit, HRV, MWL, MAT, NASA-TXL	AHR reading for MWL was 67 BPM for no task and 78 for MAT task	Heart rate (BPM)
Diaz-Piedra et al. (2020)	DS, theta-EEG, MWL, terrain complexities	Real-time monitoring of cognitive states and improving road safety in military and civilian contexts	Theta EEG, MWL, combat and non-combat driving scenarios
Wang et al. (2021)	Cohort study, HRV, CVD, SDNN, RMSSD	SDNN and LF levels are, independent predictors of CVD and hypertensive disease, also useful for predicting 8-year CVD risk	Hypertensive disease, congestive heart failure, CHF
Wang et al. (2010)	Dataset, HRV, K-NN, PCA	The HRV-parameter-based recognition strategy achieved the best performance among three recognition methods	HRV
Mahachandra et al. (2012)	DS, HRV, EEG, RMSSD	These biological signals can be considered in developing sleepiness detection system	HRV, EEG
Ma and Feng (2023)	AVP, UX, MWL	Scenario-based explanations improved situational trust, UX, and MWL of drivers	Situational trust, user experience, MWL, reaction time, return times
Persson et al. (2020)	DS, HRV, K-NN, SVM	Random forest classifier achieved the best binary classification performance	RMSSD, NN50, pNN50, mean NN, and SSD1. AdaBoost, random forest
Shakouri et al. (2018)	DS, RMSSD, MANCOVA, NASA-TLX	There were weak correlations between heart rate variability measures	CML, JLM, HRV, LF, HF, RR

### 4.2 Indicators of MWL used in the driving context. Part-2

We have explored various methods and their impact on driving performance and workload. Notable findings include improved driving performance with specific interventions (e.g., DS, AOSPAN, PASAT), safety consequences during obstacle encounters (F&Tt, AdaptIVe), and the influence of lighting conditions (DS, F&Tt, EEG). Metrics such as heart rate, eye movement, and pupillary response were used to assess workload. Additionally, SVM demonstrated high accuracy in workload classification tasks. Overall, these studies contribute valuable insights for enhancing driving safety and efficiency. In this comprehensive investigation encompassing diverse methodologies, in we have meticulously scrutinized critical parameters associated with driving performance. Notably, EEG theta power activity emerged as a robust workload indicator among army combat drivers, holding significant promise for real-world training scenarios. The integration of wearable sensor technologies, specifically around-the-ear electrode arrays, presents an avenue for real-time monitoring of cognitive states, thereby enhancing road safety. Furthermore, in Table 2 the study delved into cardiovascular health, identifying independent predictors of cardiovascular disease (CVD) and hypertensive disease through parameters such as standard deviation of normal-to-normal intervals (SDNN) and low-frequency (LF) levels. These insights hold valuable implications for assessing long-term CVD risk. The heart rate variability parameter-based recognition strategy outperformed alternative methods, positioning it as a valuable tool for identifying intricate patterns within heart rate variability. Additionally, biological signals, such as root mean square of successive RR interval differences (RMSSD) and standard deviation of Poincaré plot (SD1), exhibited sensitivity in detecting sleepiness, offering potential for the development of effective sleepiness detection systems.

**Table 2:** Type of study used in this research paper, the main parameters measured, computational tool and the driving task allocated during the study.

**Article**	**Type of study**					**Main parameters measured**								**Computational tools**				**Automotive task**			
	**Driving simulator**	**Field and track**	**Literature review**	**Data set**	**Case study**	**Heart rate**	**Eye**	**EEG**	**ECG**	**EMG**	**Blood flow**	**Hypertension**	**Reaction time**	**Machine learning**	**HardWare**	**Statistical tools**	**Automotive tools**	**LKA**	**CKM**	**JLM**	**TOR**
Chen et al. (2021)	✓					✓	✓	✓								✓	✓				✓
Rösener et al. (2017)		✓					✓										✓			✓	
Noubissie Tientcheu et al. (2022)			✓					✓								✓	✓				✓
Figalová et al. (2022)	✓					✓										✓	✓	✓			
Kajiwara and Murata (2022)	✓						✓									✓	✓			✓	✓
Perozzi et al. (2022)			✓			✓		✓								✓	✓				✓
Eichelberger and McCartt (2015)	✓					✓						✓				✓				✓	
Park et al. (2020)			✓			✓							✓								✓
Murtaza et al. (2023a)		✓						✓	✓					✓	✓	✓					✓
Murtaza et al. (2024a)		✓								✓			✓				✓	✓			✓
Marquart et al. (2015)	✓							✓	✓	✓	✓				✓		✓				✓
Chen et al. (2022)	✓										✓			✓				✓			✓
Yoon and Ji (2019)	✓					✓		✓	✓					✓					✓		
Ko and Ji (2018)	✓	✓											✓			✓		✓			
Li et al. (2020)		✓				✓									✓	✓	✓				✓
Kanakapura Sriranga et al. (2023)			✓			✓		✓	✓	✓				✓					✓		✓
Islam et al. (2020)			✓			✓		✓	✓	✓					✓		✓			✓	
Cardone et al. (2022)	✓					✓		✓	✓	✓				✓			✓				✓
Biondi et al. (2017)	✓	✓				✓								✓	✓			✓			
Lu et al. (2022)					✓											✓					✓
Piechulla et al. (2003)	✓							✓	✓	✓						✓	✓		✓		
Li and Chung (2013)					✓	✓		✓	✓	✓		✓				✓		✓			✓
Makhtar and Sulaiman (2020)				✓		✓										✓		✓			
Diaz-Piedra et al. (2020)	✓					✓		✓	✓							✓	✓				✓
Wang et al. (2021)					✓						✓		✓			✓	✓	✓			
Wang et al. (2010)			✓													✓	✓				✓
Mahachandra et al. (2012)				✓										✓					✓		
Ma and Feng (2023)	✓															✓	✓				✓
Persson et al. (2020)				✓										✓					✓		
Shakouri et al. (2018)	✓															✓	✓				
∑	14	6	6	3	3	14	3	12	9	7	3	2	4	8	5	18	17	8	5	4	17

**Figure 2:**
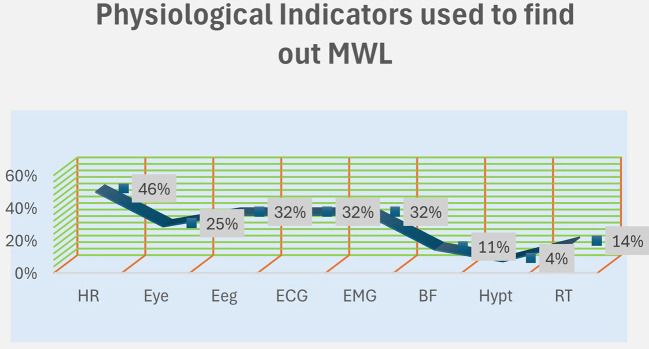
Highlights the relative significance of these parameters within the studied domain, emphasizing heart rate and eye-related metrics measurements as key factors.

**Figure 3:**
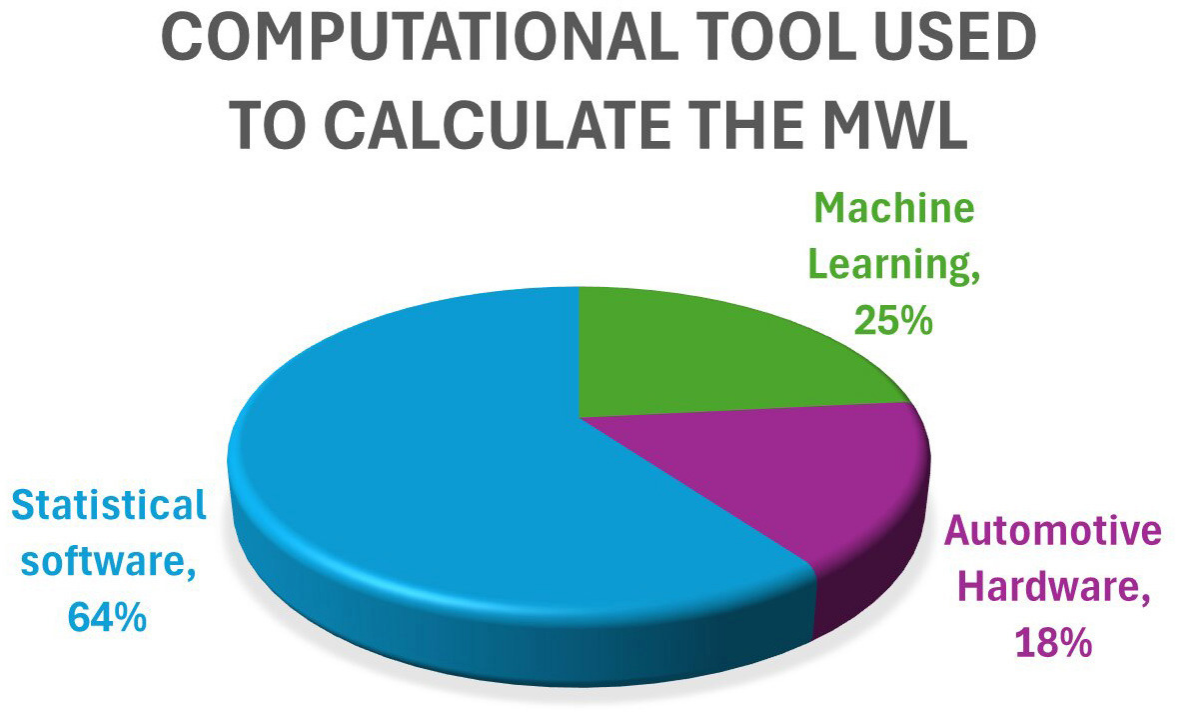
The impact of different computational tools and a breakdown of their contributions used in this study.

In this specific scenario, an investigation is carried out concerning the impacts of different physiological parameters. In Figure 2 a comprehensive examination is performed regarding their individual contributions such as HR, Eye, ECG, and other parameters. Heart Rate (HR): With the most significant weightage of 46%, heart rate occupies a central position within the physiological framework. It potentially serves as an indicator of an individual's cardiovascular health or stress levels, thus representing a crucial aspect of their overall wellbeing. Eye: Holding a substantial 25% contribution, parameters related to the eye are important. These parameters may involve visual attention, eye movements, or cognitive processes, offering insights into various cognitive and visual functions. Electroencephalogram (EEG), Electrocardiogram (ECG), and Electromyogram (EMG): Each of these factors carries a 32% contribution, emphasizing their equal importance in the realm of physiology. EEG records the brain's electrical activity, ECG monitors heart function, while EMG assesses muscle activity levels, collectively providing essential insights into diverse bodily functions. Blood Flow (BF): Representing 11% of the overall impact, blood flow may seem less prominent compared to other parameters. Nevertheless, its interpretation within a particular context could yield valuable and detailed information concerning physiological responses. Hypertension (Hypt): Contributing minimally at only 4%, hypertension appears to exert a relatively minor influence on the ongoing physiological assessment. Reaction Time (RT): Playing a moderate role with a 14% contribution, reaction time introduces another layer to the thorough analysis of physiological parameters, underscoring the significance of prompt responses in various situations. Figure 3 illustrates the predominant utilization of computational tools in the calculation of physiological parameters. Statistical software, accounting for 64%, is primarily employed for data analysis and hypothesis testing. Machine learning techniques, representing 25%, play a significant role in predictive modeling and pattern recognition. Additionally, automotive hardware tools, constituting 18%, are crucial for vehicle control and performance optimization.

In summary the paper presented a thorough examination of the cognitive deficits resulting from mental workload and heart rate variability within the driving safety framework, emphasizing the relationship among cognitive impairment, workload, and physiological signals such as heart rate and eye movements. The research underscores the significance of understanding the interplay among cognitive abilities, workload requirements, and bodily reactions for ensuring driving safety. One of the limitations of the current research is the lack of consideration for individual differences in cognitive and physiological responses to mental workload. Age, driving experience, and cognitive abilities may influence how drivers perceive and respond to cognitive demands. Future studies should investigate these individual differences to develop more personalized and adaptive interventions for enhancing driving safety. Another limitation is the reliance on simulated driving environments in many reviewed studies. While driving simulators provide a controlled setting for assessing driver performance and cognitive states, they may not fully capture the complexities and unpredictability of real-world driving situations. Future research should aim to validate the findings from simulator studies in real-world driving conditions to ensure the generalizability and ecological validity of the results. Despite these limitations, the findings from this review have important implications for the development of driver monitoring systems and the design of adaptive interfaces. By incorporating physiological measures such as HRV along with ML techniques, it is possible to create more effective and personalized interventions for managing driver mental workload and preventing cognitive impairment. However, successfully implementing these approaches will require close collaboration between researchers, industry partners, and policymakers to develop reliable, valid, and ethically sound solutions.

## 5 Conclusion and future work

The outcomes derived from this extensive analysis underscore the intricate relationship between mental workload, cognitive impairments, and physiological indicators within the context of driving safety. The results indicate that assessments of HRV are essential for measuring fatigue and workload, particularly in experienced drivers. This assertion is consistent with previous research that has identified HRV as a significant parameter for evaluating mental workload and cognitive stress. However, these limitations restrict the applicability of the results to actual driving scenarios and may neglect critical individual variances in cognitive and physiological reactions to mental workload. Subsequent investigations should concentrate on several pivotal domains to mitigate these limitations and propel the discipline forward. Initially, research should endeavor to corroborate the outcomes from simulator investigations in authentic driving environments to ensure the ecological validity of the findings. This may necessitate the innovation of new methodologies and the utilization of sophisticated sensor technologies to capture performance and cognitive states in real-world contexts. Secondly, future inquiries ought to explore individual variations in cognitive and physiological reactions to mental workload, taking into account variables such as age, experience, and cognitive capabilities. This will facilitate the creation of more tailored and adaptive strategies for improving safety. Thirdly, there is a pressing need for enhanced standardization in research methodologies and the establishment of validated instruments for evaluating mental workload and cognitive deficits. This will promote the comparison of results across studies and the recognition of substantial patterns and trends. Lastly, collaboration among researchers, industry stakeholders, and policymakers is essential for translating research outcomes into practical implementations. This could involve formulating guidelines for the design and deployment of systems and instituting standards for data collection, analysis, and interpretation. Further empirical inquiry is warranted to investigate the incorporation of supplementary indicators of cognitive workload, including subjective evaluations or task performance metrics, to attain a more holistic comprehension of mental workload and its implications for cognitive deficits. Subsequent research could analyze the efficacy of interventions or technological solutions intended to mitigate cognitive deficits resulting from mental workload. This may involve the creation and validation of pioneering technologies capable of real-time monitoring and management of mental workload *in situ*. There exists a pressing necessity for investigations that assess the transferability of the results beyond the particular context examined. Examination could be conducted to elucidate the ramifications of mental workload and cognitive deficits in additional domains such as healthcare or aviation, aiming to elucidate broader implications and potential remedial measures. Longitudinal studies could be executed to evaluate the enduring effects of mental workload on cognitive deficits and to pinpoint variables that might affect cognitive performance over time. Despite progress in employing physiological indicators for the evaluation of cognitive workload, obstacles persist in the standardization of methodologies and in elucidating the complex interplay between physiological markers and psychological conditions. Further investigation is imperative to explore individual differences in physiological reactions and to develop dependable systems for fatigue detection that can be applied in practical settings. The integration of multiple assessment modalities, such as physiological sensors and cognitive evaluations, can enhance the precision and dependability of mental workload assessments.
